# Using SCC Antigen and CRP Levels as Prognostic Biomarkers in Recurrent Oral Cavity Squamous Cell Carcinoma

**DOI:** 10.1371/journal.pone.0103265

**Published:** 2014-07-25

**Authors:** I-How Chen, Chun-Ta Liao, Hung-Ming Wang, Jung-Ju Huang, Chung-Jan Kang, Shiang-Fu Huang

**Affiliations:** 1 Department of Otolaryngology, Chang Gung Memorial Hospital and Chang Gung University, Taiwan, ROC; 2 Department of Medical Oncology, Chang Gung Memorial Hospital, Chang Gung University, Taoyuan, Taiwan, ROC; 3 Department of Plastic and Reconstructive Surgery, Chang Gung Memorial Hospital Linkou Medical Center and Chang Gung University, Taiwan, ROC; Duke Cancer Institute, United States of America

## Abstract

Squamous cell carcinoma antigen (SCC-Ag) and C-reactive protein (CRP) levels have been successfully used to stratify risk groups in primary oral squamous cell carcinoma (OSCC) patients; however, related biomarkers have rarely been investigated in recurrent OSCC. The purpose of the present study was to analyze the relationships of SCC-Ag and CRP levels at the time of recurrence with clinical factors and prognosis. We retrospectively recruited patients with recurrence in a cohort of 534 OSCC patients between March 2001 and July 2013. One hundred patients had recurrence. The serum SCC-Ag and CRP levels were measured at the time of cancer diagnosis, 3 to 6 months after treatment with clinical disease-free, and at the time of recurrence. The SCC-Ag levels were significantly lowered after treatment (paired t-test: p = 0.001) and re-elevated at the time of recurrence (paired t-test: p = 0.027). An SCC-Ag level ≥2.0 ng/ml and a CRP level ≥5.0 mg/L at the time of recurrence were significantly associated with recurrent tumor status (P<0.001), recurrent nodal metastasis (χ^2^ trend test: P = 0.020), distant metastasis (P<0.001), and overall survival (P<0.001). Moreover, the influence of both elevated SCC-Ag and CRP levels on overall survival (P<0.001, H.R. [95% CI]: 5.406 [2.210–13.222]) still existed after adjusting for the recurrent tumor stage and patient age. The present study demonstrates that concurrent high levels of both SCC-Ag and CRP at the diagnosis of recurrence acts as a predictor of recurrent tumor status, recurrent advanced tumor stage, distant metastasis, and survival after the diagnosis of recurrence. This study expands the applicability of these two markers in the risk stratification in recurrent OSCC.

## Introduction

Oral cancer is one of the most common cancers worldwide and the fifth most common in Taiwan. [1] One of the primary treatments for oral squamous cell carcinoma (OSCC) is radical excision with or without adjuvant chemoradiotherapy, which has proven to be effective in locoregional control of the disease. [2] Nonetheless, the search for biomarkers involved in atypical tumor behavior may aid clinicians in the selection of appropriate treatment strategies for these patients. [3], [4] The presence of elevated SCC-Ag levels has been found to be associated with higher malignancy in terms of tumor stage, tumor thickness, and survival in OSCC patients. [4], [5] A second potentially important marker is the acute-phase protein C-reactive protein (CRP), the level of which has also been shown to correlate with survival in certain human cancers. [3], [6], [7], [8], [9] Indeed, elevated CRP levels act as a marker of chronic inflammation in the tumor microenvironment, with chronic inflammation itself acting as a stimulus for angiogenesis and cell proliferation and an inhibitor of apoptosis. [10], [11], [12] We previously demonstrated that SCC-Ag elevation in OSCC is associated with poor survival and tumor invasiveness. The relationship between SCC-Ag and CRP and their potential combined value as prognostic markers of disease-free survival have also been demonstrated in OSCC. [3], [4], [13]

Upon recurrence, the outcomes of salvage treatment in recurrent OSCC are typically disappointing: the survival rate of patients with local recurrence is 33.3%, which is significantly lower than that (94.3%) of patients without local recurrence. [14] In addition, recurrence at unusual sites, such as the retropharyngeal nodes, parotid nodes, or pre-laryngeal nodes, carries an even worse prognosis. [15] Prognostic indicators, such as neoadjuvant chemotherapy, and surgical margin status have been found to be independent risk factors, whereas few biomarkers that successfully predict disease progression and patient prognosis in recurrent OSCC have been identified. [14]

In this study, we retrospectively analyzed the roles of SCC-Ag and CRP in recurrent OSCC to test the combined prognostic value of elevated SCC-Ag and CRP levels in this group of patients.

## Patients and Methods

### Ethics statement

The study was approved by the Institutional Review Board (IRB) of Chang Gung Memorial Hospital, Chang Gung University Medical College. The data (CRP and SCC-Ag) were collected retrospectively and no informed consent was requested by the IRB. The information was recorded by the investigator in a manner that subjects were anonymized and de-identified prior to analysis.

### OSCC patients

We retrospectively reviewed the charts of 534 OSCC patients who were diagnosed with primary OSCC between March 2001 and July 2013 in our institute. There were 492 males and 42 females, with a mean age of 51.5 years (range 24 to 84 years). Patients initially diagnosed with distant metastasis or verrucous carcinoma were excluded from our study. The follow-up period for each patient began at the time of cancer diagnosis and ended at death or August 2013; the mean follow-up period was 33.53 months (range 0–136 months). The stage distributions and treatment modalities of the included OSCCs are listed in Table S1.

### Treatment of OSCC

The patients were staged according to the TNM staging system proposed by the American Joint Committee on Cancer (AJCC, 2002 edition). [16] Most of the patients underwent radical tumor excision, with or without neck dissection based on the clinical stage after the preoperative tumor survey. [4] Patients with advanced tumor stage (T3 or T4), lymph node extracapsular spread (ECS), tumor depth ≥10 mm, or poor tumor differentiation received postoperative radiotherapy or concomitant chemoradiotherapy for 4–8 weeks after surgery. [17], [18] Some patients received radical chemoradiation therapy, including one patient who underwent anti-EGFR targeted therapy. The treatment modalities are included in Table S1.

### Follow-up

All the patients were followed up periodically, consisting of a checkup every month during the first 6 months after treatment, every 2 months during the following 6 months, every 3 months during the second year, and every 6 months thereafter. At every clinic visit, the physical examination included the status of the nasopharynx, oral cavity and oropharynx, and neck. The patients were subjected to a hemogram, blood chemistry, chest X-ray, and computed tomography (CT) or magnetic resonance imaging (MRI) at 3^rd^ and 6^th^ months in the first year and then annually for the following 5 years. Patients who presented abnormal clinical symptoms/signs or laboratory data underwent a bone scan and liver ultrasound. All suspicious lesions were biopsied to prove recurrence. All patients with recurrence underwent liver ultrasound, CT, or MRI of the head and neck and a whole-body bone scan or positron emission tomography. During the follow-up period, a total 100 patients had recurrence and their SCC-Ag and CRP levels checked at the time of recurrence.

### CRP measurement

Serum CRP levels were evaluated at the time of cancer diagnosis, post-treatment 3 to 6 months with clinical disease-free, and at the time of recurrence. Serum CRP levels were measured using an auto-analyzer (Hitachi 7600-210, Hitachi Medico, Tokyo). The cut-off point for serum CRP was set at 5.0 mg/L, which is the internationally adopted value for inflammation. [3], [13]

### SCC-Ag measurement

The serum SCC-Ag level was also measured at the time of cancer diagnosis, post-treatment 3 to 6 months with clinical disease-free, and at the time of recurrence using a commercially available chemiluminescent microparticle immunoassay (CMIA) (Abbott Japan Co., Ltd., Tokyo, Japan). The reference cut-off value for the serum SCC-Ag level was set at 2.0 ng/ml, as previously established [4], [13], [19].

### Statistical analysis

The statistical methods used included a univariate analysis with the chi-square test. The univariate analysis of survival differences was calculated by the log-rank test. The overall survival (OS) was defined from the time of recurrence to the time of death or follow-up, whichever came first. A multivariate analysis of survival using Cox's proportional hazard model was performed to determine the prognostic value. The linear relationship between two variables was calculated by a linear regression analysis. A two-sided *P* value <0.05 was considered statistically significant. These analyses were performed using the Statistical Package software for the Social Sciences (SPSS), version 13.0 (SPSS, Inc., Chicago, IL, USA).

## Results

### Patient characteristics

The clinicopathological characteristics of the 534 OSCC patients (492 males and 42 females) are shown in Table S1. The tongue (N = 231, 43.3%) and the buccal mucosa (N = 200, 37.5%) were the most common primary tumor sites. The tumor stage distribution was 120 (22.5%) in stage I, 121 (22.7%) in stage II, 63 (11.8%) in stage III, 188 (35.2%) in stage IVa, and 42 (7.9%) in stage IVb. All of the primary treatment modalities are listed in Table S1.

Of the 100 patients enrolled in this study during the follow-up period, 45 had local recurrence, 16 had cervical lymph node recurrence, 11 had locoregional recurrence, 3 had local recurrence and distant metastasis, 4 had neck recurrence and distant metastasis, 17 had distant metastasis, and 4 had locoregional recurrence with distant metastasis. Thirty-nine patients died of the disease during the follow-up period. One patient died of non-cancer-related causes.

The distribution of SCC-Ag at the time of cancer diagnosis (n = 321), post-treatment (n = 318) and at recurrence (n = 100) was shown in Fig. S1A. The SCC-Ag was significantly lowered after-treatment (1.17 ng/mL, ± standard deviation (±S.D.): 0.62) when compared with that at cancer diagnosis (mean: 2.55 ng/mL, ±S.D.: 5.17) (paired t-test: p = 0.001). And the SCC-Ag levels were significantly re-elevated from post-treatment (mean: 1.17 ng/mL, ± S.D.: 0.62) to recurrence (mean: 4.07 ng/mL, ± S.D.: 15.21) (paired t-test: p = 0.027). The distribution of CRP at the time of cancer diagnosis (n = 321), post-treatment (n = 214) and at recurrence (n = 100) was shown in Fig. S1B. The CRP was not significantly different between at cancer diagnosis (mean: 7.77 mg/L, ± S.D.: 13.94) and after-treatment (5.00 mg/L, ± S.D.: 11.52) (paired t-test: p = 0.129). And the CRP levels were also insignificantly different between post-treatment (mean: 5.00 mg/L, ± S.D.: 11.52) and recurrence (mean: 24.17 mg/L, ± S.D.: 44.09) (paired t-test: p = 0.306).

### CRP levels at the time of recurrence, clinicopathological variables, and prognosis

Higher pre-treatment CRP level (CRP≥5.0 mg/L) was closely related with pathological tumor status (P<0.001), pathologic lymph node metastasis with ECS (P = 0.025), bone invasion (P<0.001), skin invasion (P<0.001) and tumor depth (>10 mm vs. ≤10 mm, P<0.001).

A close association was also observed between a high CRP level (CRP≥5.0 mg/L) and recurrent tumor status (P = 0.026), recurrent nodal metastasis (P = 0.014), distant metastasis (P<0.001), and recurrent tumor stage (P<0.001) (Table 1).

**Table 1:** Association of CRP and SCC-Ag levels at the time of recurrence (N = 100).

	CRP			SCC-Ag		
Recurrence staging	Negative [n (%)]	Positive [n (%)]	P value	Negative [n (%)]	Positive [n (%)]	P value
Recurrent tumor stage						
I (n = 11)	10 (90.9)	1 (9.1)	**<0.001**	10 (90.9)	1 (9.1)	**<0.001** *
II (n = 11)	7 (63.6)	4 (36.4)		11 (100.0)	0 (0.0)	
III (n = 7)	6 (85.7)	1 (14.3)		7 (100.0)	0 (0.0)	
IVa (n = 39)	18 (46.2)	21 (53.8)		29 (74.4)	10 (25.6)	
IVb (n = 4)	3 (75.0)	1 (25.0)		3 (75.0)	1 (25.0)	
IVc (n = 28)	6 (21.4)	22 (78.6)		10 (35.7)	18 (64.3)	
Recurrent primary tumor status						
T0 (n = 38)	13 (34.2)	25 (65.8)	**0.026**	20 (52.6)	18 (47.4)	**0.050**
T1 (n = 11)	10 (90.9)	1 (9.1)		10 (90.9)	1 (9.1)	
T2 (n = 14)	9 (64.3)	5 (35.7)		12 (85.7)	2 (14.3)	
T3 (n = 3)	2 (66.7)	1 (33.3)		3 (100.0)	0 (0.0)	
T4a (n = 28)	13 (46.4)	15 (53.6)		21 (75.0)	7 (25.0)	
T4b (n = 6)	3 (50.0)	3 (50.0)		4 (66.7)	2 (33.3)	
Recurrent nodal status						
N0 (n = 63)	34 (54.0)	29 (46.0)	**0.014**	49 (77.8)	14 (22.2)	**0.007**
N1 (n = 5)	5 (100.0)	0 (0.0)		5 (100.0)	0 (0.0)	
N2 (n = 32)	11 (34.4)	21 (65.6)		16 (50.0)	16 (50.0)	
Distant metastasis						
No (n = 72)	44 (61.1)	28 (38.9)	**<0.001**	60 (83.3)	12 (16.7)	**<0.001**
Yes (n = 28)	6 (21.4)	22 (78.6)		10 (35.7)	18 (64.3)	

^*^Fisher's exact test.

**Figure 1:**
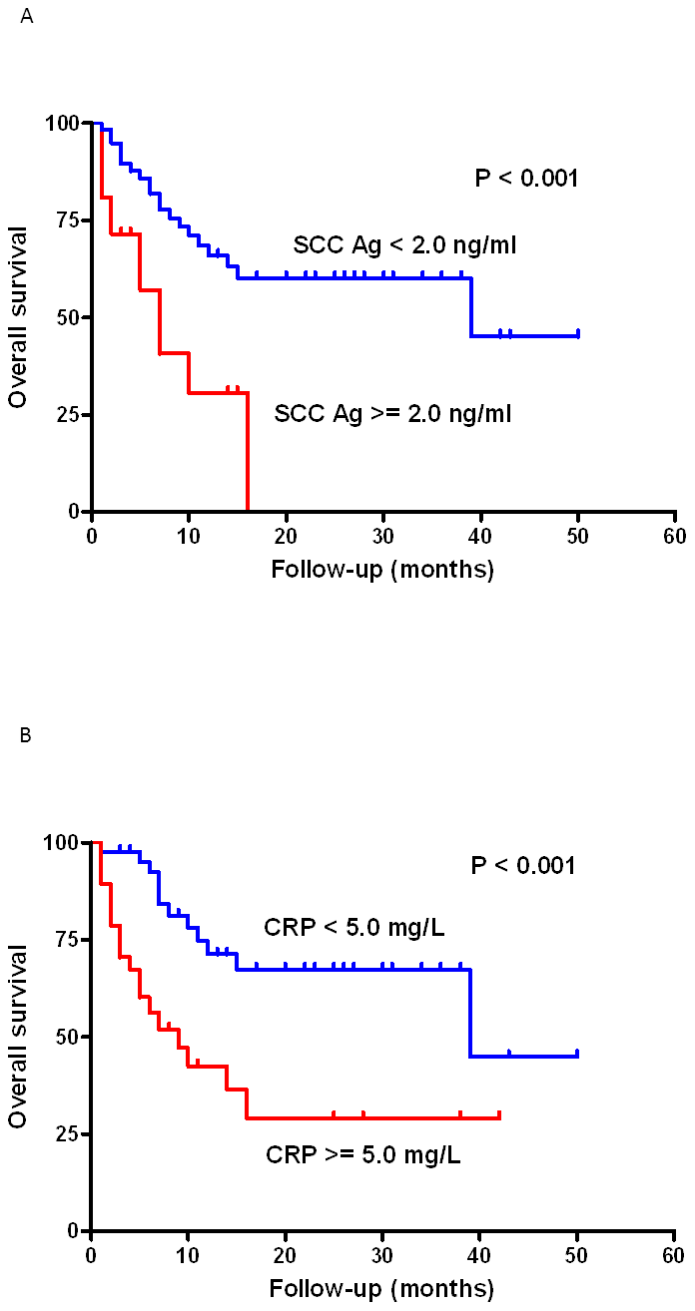
Survival curves in 100 recurrent OSCC patients according to (A) CRP level (log rank test, P<0.001) and (B) SCC-Ag level (log-rank test, P = 0.001).

When the survival rates of the high CRP group (CRP≥5.0 mg/L) and the low CRP group (CRP<5.0 mg/L) were compared, overall survival (OS) was significantly lower in the high CRP group than the low CRP group (log-rank test, P<0.001, Fig. 1A).

### SCC-Ag level at the time of recurrence and its relationship with clinicopathological variables and prognosis

All the patients with higher SCC-Ag level (SCC-Ag ≥2.0 ng/ml) before treatment had higher risks of advanced pathological tumor status (P<0.001), pathologic nodal metastasis lymph node ECS (P = 0.004), bone invasion (P<0.001), skin invasion (P<0.001) and tumor depth (>10 mm vs. ≤10 mm, P<0.001).

A close association was also observed between a high SCC-Ag level (SCC-Ag ≥2.0 ng/ml) and recurrent tumor status (P = 0.050), recurrent nodal metastasis (P = 0.007), distant metastasis (P<0.001), and recurrent tumor stage (P<0.001) (Table 1).

**Figure 2:**
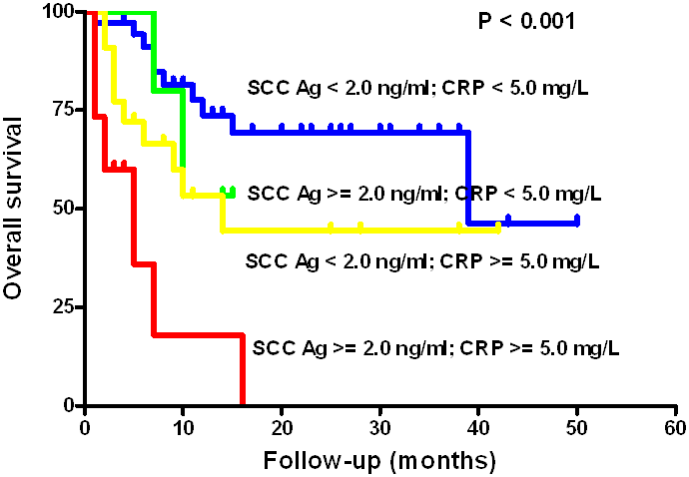
Survival curves in 100 recurrent OSCC patients related to the CRP and SCC-Ag levels. The low CRP and low SCC-Ag group showed significantly better OS compared to the high CRP and high SCC-Ag group (P<0.001).

The survival rates of the high SCC-Ag level group (SCC-Ag ≥2.0 ng/ml) and low SCC-Ag level group (SCC-Ag <2.0 ng/ml) were compared, and OS was found to be significantly lower in the high SCC-Ag group (log-rank test, P = 0.001, Fig. 2).

### Combined CRP and SCC-Ag level and its relationship with clinicopathological variables and prognosis

The patients were divided into four groups according to the presence of high or low SCC-Ag and CRP levels, and a close association was observed between the coexistence of high SCC-Ag (≥2.0 ng/ml) and high CRP (≥5.0 mg/L) and recurrent tumor status (P<0.001), recurrent nodal metastasis (χ^2^ trend test: P = 0.020), and distant metastasis (P<0.001) (Table 2).

**Table 2:** Association of CRP and SCC-Ag levels at the time of recurrence (N = 100).

	CRP (−), SCC-Ag (−)	CRP (−), SCC-Ag (+)	CRP (+), SCC-Ag (−)	CRP (+), SCC-Ag (+)	
	[n (%)]	[n (%)]	[n (%)]	[n (%)]	P value
Recurrent tumor stage					
I (n = 11)	9 (81.8)	1 (9.1)	1 (9.1)	0 (0.0)	**<0.001**
II (n = 11)	7 (63.6)	0 (0.0)	4 (36.4)	0 (0.0)	
III (n = 7)	6 (85.7)	0 (0.0)	1 (14.3)	0 (0.0)	
IVa (n = 39)	15 (38.5)	3 (7.7)	14 (35.9)	7 (17.9)	
IVb (n = 4)	2 (50.0)	1 (25.0)	1 (25.0)	0 (0.0)	
IVc(n = 28)	3 (10.7)	3 (10.7)	7 (25.0)	15 (53.6)	
Recurrent primary tumor status					
T0 (n = 38)	11 (28.9)	2 (5.3)	9 (23.7)	16 (42.1)	**0.064**
T1 (n = 11)	9 (81.8)	1 (9.1)	1 (9.1)	0 (0.0)	
T2 (n = 14)	8 (57.1)	1 (7.1)	4 (28.6)	1 (7.1)	
T3 (n = 3)	2 (66.7)	0 (0.0)	1 (33.3)	0 (0.0)	
T4a (n = 28)	10 (35.7)	3 (10.7)	11 (39.3)	4 (14.3)	
T4b (n = 6)	2 (33.3)	1 (16.7)	2 (33.3)	1 (16.7)	
Recurrent nodal status					
N0 (n = 63)	29 (46.0)	5 (7.9)	20 (31.7)	9 (14.3)	**0.011**
N1 (n = 5)	5 (100.0)	0 (0.0)	0 (0.0)	0 (0.0)	
N2 (n = 32)	8 (25.0)	3 (9.4)	8 (25.0)	13 (40.6)	
Distant metastasis					
No (n = 72)	39 (54.2)	5 (6.9)	21 (29.2)	7 (9.7)	**<0.001**
Yes (n = 28)	3 (10.7)	3 (10.7)	7 (25.0)	15 (53.6)	

CRP (−), CRP level <5.0 mg/L; CRP (+), CRP level ≥5.0 mg/L; SCC-Ag (−), SCC-Ag <2.0 ng/ml; SCC-Ag (+), SCC-Ag ≥2.0 ng/ml.

When the survival rates of the four groups were compared, the OS of the high SCC-Ag and high CRP group was significantly lower than that of the other groups (log-rank test, P<0.001, Figure 2, Table 3). The clinicopathological factors that influence patient OS after the diagnosis of recurrence were evaluated in univariate analyses. Recurrent primary tumor status (P = 0.035), recurrent lymph node metastasis (P = 0.003), high CRP level (≥5.0 mg/L) (P<0.001), high SCC-Ag level (≥2.0 ng/ml) (P = 0.001), and distant metastasis (P<0.001) were found to significantly influence OS after recurrence (Table 3).

**Table 3:** Univariate log-rank test of prognostic covariates in 100 patients with oral cavity squamous cell carcinoma regarding their overall survival after the diagnosis of recurrence.

Characteristics (n,%)	Mean survival (months)	P value	HR (95% CI)
Age (years) of recurrence			
<50 (46, 46%)	25.90	0.550	1
≥50 (54, 54%)	22.88		1.214 (0.643–2.293)
Sex			
Female (8, 8%)	27.24	0.095	1
Male (92, 92%)	7.44		2.116 (0.879–5.097)
Recurrent tumor status			
T1–2 (25, 40.3%)	40.27	**0.035**	1
T3–4 (37, 59.7%)	22.11		3.326 (1.088–10.173)
Recurrent nodal status			
(−) metastasis (63, 63%)	32.78	**0.003**	1
(+) metastasis (37, 37%)	14.35		2.594 (1.379–4.877)
Distant metastasis			
No (72, 72%)	31.44	**<0.001**	1
Yes (28, 28%)	4.02		7.024 (3.360–14.687)
Recurrent stage			
I–II (22, 22%)	43.96	**<0.001**	1
III–IV (78, 78%)	17.48		2.898 (1.547–5.427)
CRP			
<5 mg/ml (50, 50%)	33.49	**<0.001**	1
≥5 mg/ml (50, 50%)	14.72		3.580 (1.815–7.061)
SCC-Ag			
<2 ng/ml (70, 70%)	30.20	**0.001**	1
≥2 ng/ml (30, 30%)	7.70		3.185 (1.639–6.187)
SCC-Ag and CRP		**<0.001**	
SCC-Ag <2 ng/ml, CRP<5 mg/L (42, 42%)	34.95		1
SCC-Ag ≥2 ng/ml, CRP<5 mg/L (8, 8%)	10.56	0.219	2.261 (0.616–8.296)
SCC-Ag <2 ng/ml, CRP≥5 mg/L (28, 28%)	19.89	**0.011**	2.948 (1.285–6.762)
SCC-Ag ≥2 ng/ml, CRP≥5 mg/L (22, 22%)	5.07	**<0.001**	7.913 (3.283–19.073)

CRP (−), CRP level <5.0 mg/L; CRP (+), CRP level ≥5.0 mg/L; SCC-Ag (−), SCC-Ag <2.0 ng/ml; SCC-Ag (+), SCC-Ag ≥2.0 ng/ml.

Patients with recurrence could have local disease, nodal metastasis or distant metastasis concurrently at the time of diagnosis. To eliminate the possible interaction between the variables, we put overall stage instead of tumor status, nodal status and distant metastasis separately into multivariate analysis. In Table 4, multivariate analysis indicated that high SCC-Ag (≥2.0 ng/ml) and CRP (≥5.0 mg/L) levels significantly influenced OS after recurrence (P<0.001, H.R. [95% CI]: 5.406 [2.210–13.222]) after adjusting for the recurrent tumor stage and patient age. These results indicate that the combination of preoperative SCC-Ag and CRP levels is an independent prognostic indicator in patients with recurrent OSCC.

**Table 4:** Multivariate Cox regression model of prognostic covariates in 100 patients with oral cavity squamous cell carcinoma regarding their disease-free and overall survival.

	OS	
Characteristic	P value	HR (95% CI)
Age (years)		
<50	0.521	1
≥50		1.243 (0.640–2.414)
Recurrent tumor stage		
Early^a^	**0.003**	1
Advanced^b^		2.694 (1.416–5.124)
SCC-Ag and CRP	**0.003**	
SCC-Ag <2 ng/ml, CRP<5 mg/L		1
SCC-Ag ≥2 ng/ml, CRP<5 mg/L	0.347	1.883 (0.504–7.038)
SCC-Ag <2 ng/ml, CRP≥5 mg/L	**0.015**	2.940 (1.237–6.988)
SCC-Ag ≥2 ng/ml, CRP≥5 mg/L	**<0.001**	5.406 (2.210–13.222)

HR, hazard ratio; CI, confidence interval.

a stages I and II, ^b^stages III, IVa, IVb, and IVc.

OS, overall survival.

## Discussion

Squamous cell carcinoma antigen (SCC-Ag) is a tumor-associated protein that was first identified in the uterine cervix by Kato and Torigoe. [20] SCC-Ag is a generic name for a homologous class of serine proteinase inhibitors (serpins) for which a number of encoding genes, including SCC-Ag 1 and SCC-Ag 2, have been identified. [21] SCC-Ag promotes tumorigenesis by inhibiting apoptosis or increasing cell migration after stimulation by epidermal growth factor and plays a role in tumor invasion and metastasis.

SCC-Ag has been clinically correlated with human cancers, particularly cervical cancer, in a number of studies. [22], [23] Moreover, the serum SCC-Ag level is useful for predicting early recurrence in OSCC, [4], [13] which may be related to the molecular role of SCC-Ag in protecting against tumor cell apoptosis. Furthermore, the pro-invasive ability of SCC-Ag has been supported by clinical studies demonstrating positive relationships between serum SCC-Ag levels and tumor progression, lymph node metastasis, and tumor stage. [4], [13], [19], [24], [25], [26] In our study, we demonstrated that the SCC-Ag was significantly lower after treatment in OSCC (Fig. S1A). Some patients with higher SCC-Ag after treatment, but they returned to be within normal range after follow-up. But it alerts clinicians the possibilities of disease progression. It was elevated when the disease was proved to have recurrence or progression. These provide evidences that SCC-Ag could be a marker that closely related to tumor status.

The present study further confirms positive relationships between the SCC-Ag level and recurrent primary tumor status, recurrent node metastasis, distant metastasis, and tumor stage (P = 0.026, P = 0.014, P<0.001, and P<0.001, respectively) (Table 1).

Although the role of CRP in oral cavity SCC is controversial, studies have recently demonstrated the prognostic value of CRP in OSCC. [27], [28], [29] CRP elevation indicates a host immune response to tumor growth, with elevated inflammatory cytokines and subsequent CRP elevation. [30] In the tumor microenvironment, pro-inflammatory cytokines, such as interleukin-6 (IL-6), IL-8, and tumor necrosis factor, lead to inflammation and angiogenesis, which subsequently up-regulate the acute-phase reactant CRP. [11], [31] IL-6 also indirectly helps CRP bind to tumor cells, which may lead to tumor cell lysis. [32] Thus, CRP is not only a response to the tumor microenvironment but may also be a reflection of tumor cell killing and local tissue damage. [3], [33]

In our previous studies, we have clearly demonstrated that elevated serum CRP is associated with advanced tumor status, skin invasion, and bone invasion and have shown significant correlations between the CRP level, DFS, and OS. From Figure S1B, the CRP levels were not significantly different between tests at cancer diagnosis and post-treatment. The elevation of CRP after treatment originates from the dental infection, mucositis or pulmonary infection. In the present study, CRP was found to be closely related to the recurrent primary tumor status, recurrent lymph node metastasis, distant metastasis, and recurrent tumor stage (P = 0.050, P = 0.007, P<0.001, and P<0.001, respectively). Patients with recurrent oral cavity cancer typically present superimposed lung infection, which further elevates the serum CRP level. According to our analysis, a persistently high CRP level in patients with recurrent disease usually indicates a poor physical condition complicated with infectious processes and results in poor prognosis (Figure 1B).

As shown in Table 2, it demonstrated the distribution of SCC-Ag and CRP in patients with distant metastases. Most of the patients with distant metastasis had both elevated SCC-Ag and CRP levels. For patients with persistent bone pain, SCC-Ag and CRP levels could aid diagnosis. Additionally, in cases of multiple lung nodules for which infection or metastasis cannot be confirmed, the serum levels of SCC-Ag and CRP could help clinicians choose the appropriate management. It is noticeable that some patients had their SCC-Ag within the normal range. In 3 of our patients with isolated skin carcinomatosis, SCC-Ags were 0.5, 1.6 and 2.2 ng/ml at the time of diagnosis. Solitary metastasis to a lung nodule was also not associated with an increase in SCC-Ag levels: two of our patients with a solitary lung metastasis had SCC-Ag levels as 0.6 and 0.8 ng/ml at the time of diagnosis. The SCC-Ag was level was elevated until the metastases involved multiple sites and the increase of SCC-Ag was related to the tumor volume and also depends on the release of SCC-Ag into the bloodstream. Isolated skin carcinomatosis and solitary lung metastasis are related to the small tumor size and not revealed by serum SCC-Ag.

To our knowledge, this is the first study to demonstrate the relationship between CRP and SCC-Ag levels in recurrent OSCC. The subgroup with high levels of both SCC-Ag and CRP exhibited significantly worse OS following recurrence compared to the other groups (P value <0.001, Figure 2). Furthermore, the combined effect of SCC-Ag and CRP on survival and their prognostic value were confirmed in a multivariate analysis after adjusting for age and recurrent tumor stage (Table 4). Together, these results indicate that the combination of SCC-Ag and CRP levels is an independent prognostic indicator in recurrent OSCC patients. In the present study, all patients with elevated SCC-Ag and CRP levels progressed and expired within 16 months of the diagnosis of recurrence.

We have previously demonstrated that combined SCC-Ag and CRP levels are an independent prognostic marker in OSCC, and this parameter may be a biomarker for predicting prognosis and stratifying patients for adjuvant therapies in the absence of traditional indications, such as lymph node ECS. [13], [19] The present study has further demonstrated the successful use of SCC-Ag and CRP for predicting survival in recurrent OSCC patients.

## Supporting Information

Figure S1 **(A) The distribution of SCC-Ag levels at the time of diagnosis of cancer (mean: 2.55 ng/mL, ± S.D.: 5.17), post-treatment (1.17 ng/mL, ± ± S.D.: 0.62) and at recurrence (mean: 4.07 ng/mL, ± S.D.: 15.21).** The red lines denote the mean of SCC-Ag levels in 3 different time. (B) The distribution of CRP levels at the time of diagnosis of cancer (mean: 7.77 mg/L, ± S.D.: 13.94), post-treatment (5.00 mg/L, ± S.D.: 11.52) and at recurrence (mean: 24.17 mg/L, ± S.D.: 44.09). The red lines denote the mean of CRP levels in 3 different time.
(ZIP)

Table S1 **Characteristics of the 534 oral cavity squamous cell carcinoma patients.**
(DOCX)
