# Supplementary figures and images for: Using SCC Antigen and CRP Levels as Prognostic Biomarkers in Recurrent Oral Cavity Squamous Cell Carcinoma
Source: PLoS One. 2014 Jul 25;9(7):e103265. doi: 10.1371/journal.pone.0103265 (PMC4111511; doi:10.1371/journal.pone.0103265)

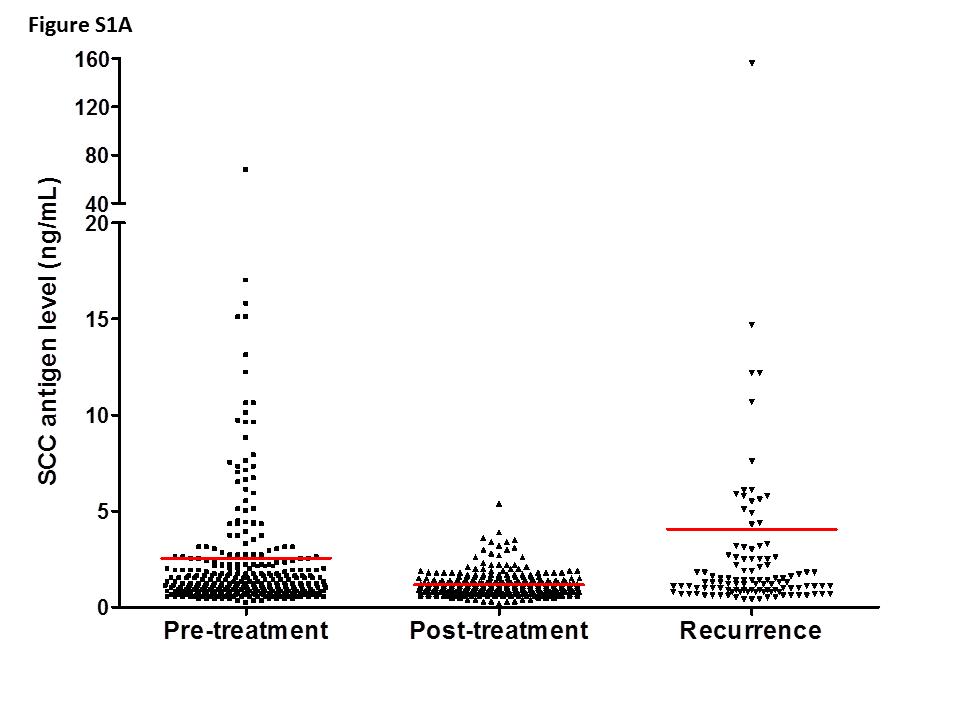

Supplement: Figure S1 — (A) The distribution of SCC-Ag levels at the time of diagnosis of cancer (mean: 2.55 ng/mL, ± S.D.: 5.17), post-treatment (1.17 ng/mL, ± ± S.D.: 0.62) and at recurrence (mean: 4.07 ng/mL, ± S.D.: 15.21). The red lines denote the mean of SCC-Ag levels in 3 different time. (B) The distribution of CRP levels at the time of diagnosis of cancer (mean: 7.77 mg/L, ± S.D.: 13.94), post-treatment (5.00 mg/L, ± S.D.: 11.52) and at recurrence (mean: 24.17 mg/L, ± S.D.: 44.09). The red lines denote the mean of CRP levels in 3 different time. (ZIP) [file pone.0103265.s001.zip › Figure S1A.tif]

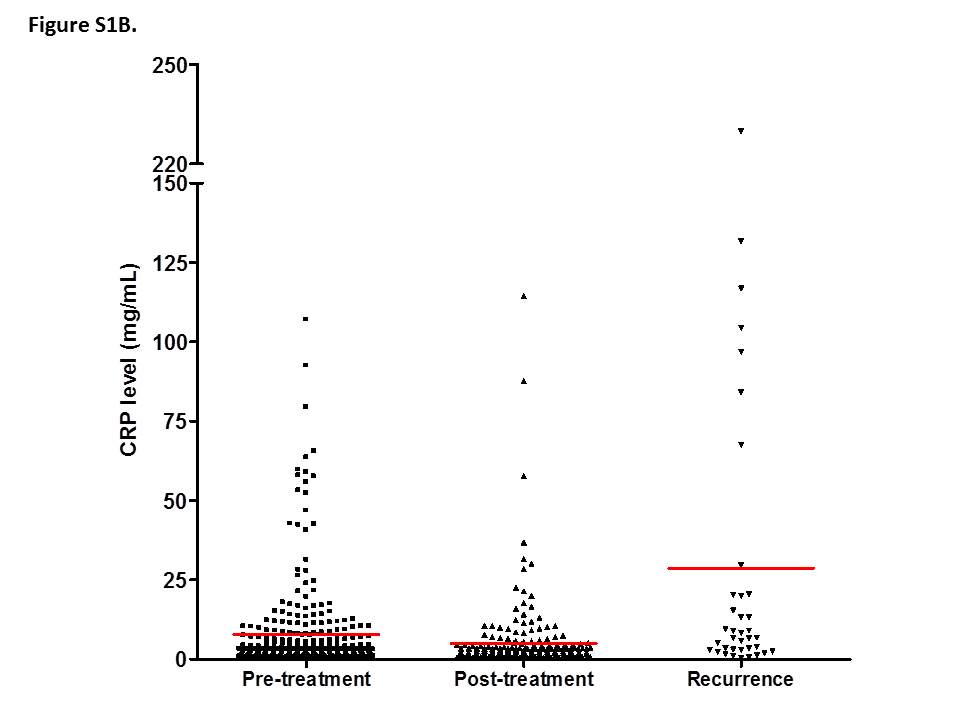

Supplement: Figure S1 — (A) The distribution of SCC-Ag levels at the time of diagnosis of cancer (mean: 2.55 ng/mL, ± S.D.: 5.17), post-treatment (1.17 ng/mL, ± ± S.D.: 0.62) and at recurrence (mean: 4.07 ng/mL, ± S.D.: 15.21). The red lines denote the mean of SCC-Ag levels in 3 different time. (B) The distribution of CRP levels at the time of diagnosis of cancer (mean: 7.77 mg/L, ± S.D.: 13.94), post-treatment (5.00 mg/L, ± S.D.: 11.52) and at recurrence (mean: 24.17 mg/L, ± S.D.: 44.09). The red lines denote the mean of CRP levels in 3 different time. (ZIP) [file pone.0103265.s001.zip › Figure S1B.tif]
