# Supplementary material for: Using SCC Antigen and CRP Levels as Prognostic Biomarkers in Recurrent Oral Cavity Squamous Cell Carcinoma
Source: PLoS One. 2014 Jul 25;9(7):e103265. doi: 10.1371/journal.pone.0103265 (PMC4111511; doi:10.1371/journal.pone.0103265)
Supplement: Table S1 — Characteristics of the 534 oral cavity squamous cell carcinoma patients. (DOCX) [file pone.0103265.s002.docx]

**Supplementary Table 1.** Characteristics of the 534 oral cavity squamous cell carcinoma patients

| Characteristic | *[No. of patients (%)]* |
| --- | --- |
| *Age (years)* |  |
| Mean (± standard deviation) | 51.5 (±11.1) |
| Range | 24.0-84.0 |
| *Gender* |  |
| Male | 492 (92.1) |
| Female | 42 (7.9) |
| *Site of primary tumor [No. of patients (%)]* |  |
| Tongue | 231 (43.3) |
| Mouth floor | 22 (4.1) |
| Lip | 12 (2.2) |
| Buccal mucosa | 200 (37.5) |
| Alveolar ridge | 38 (7.1) |
| Hard palate | 11 (2.1) |
| Retromolar trigone | 20 (3.7) |
| *Pathologic tumor status* |  |
| T1 | 134 (25.1) |
| T2 | 181 (33.9) |
| T3 | 44 (8.2) |
| T4a | 133 (24.9) |
| T4b | 42 (7.9) |
| *Pathologic N stage* |  |
| N0 | 346 (64.8) |
| N1 | 61 (11.4) |
| N2a | 1 (0.2) |
| N2b | 100 (18.7) |
| N2c | 25 (4.7) |
| N3 | 1 (0.2) |
| *Pathologic stage* |  |
| Stage I | 120 (22.5) |
| Stage II | 121 (22.7) |
| Stage III | 63 (11.8) |
| Stage IVa | 188 (35.2) |
| Stage IVb | 42 (7.9) |
| *Treatment mode* |  |
| Surgery alone | 254 (47.6) |
| Surgery with adjuvant radiation therapy | 74 (13.9) |
| Induction chemotherapy with chemoradiation therapy | 11 (2.1) |
| Induction chemotherapy with surgery and adjuvant chemoradiation therapy | 1 (0.2) |
| Concurrent chemoradiation therapy | 19 (3.6) |
| Surgery with adjuvant chemoradiation therapy | 173 (32.4) |
| Chemoradiation therapy with surgery | 2 (0.4) |
